# Towards routine biomechanical data collection in stroke rehabilitation: a usability comparison of IMU and markerless motion capture systems for functional upper-limb assessments

**DOI:** 10.1186/s12984-026-02075-7

**Published:** 2026-07-07

**Authors:** Tim Unger, Xin Yao, Anna Schmitt, Lea Cebulla, Chris Easthope Awai

**Affiliations:** 1 Data Analytics & Rehabilitation Technology (DART), Lake Lucerne Institute, Rubistrasse 9, 6354 Vitznau, Lucerne Switzerland; 2 Rehabilitation Engineering Lab, ETH Zurich, Gloriastrasse 37/39, 8092 Zurich, Switzerland; 3 Neuro-X Institute, EPFL, Campus Biotech Chemin des Mines 9, 1202 Geneva, Switzerland

**Keywords:** Usability, Neurorehabilitation, ARAT, IMU, Motion capture, Clinical routine, Markerless motion capture, Upper limb assessment

## Abstract

Objective measurement of upper-limb movement quality based on biomechanical data collected in clinical routine has the potential to enable precision neurorehabilitation at scale. However, integrating biomechanical data collection into daily clinical workflows remains challenging. In this exploratory study, we evaluated the usability of two technologies for routine kinematic data collection: an IMU-based version of the instrumented Action Research Arm Test (iARAT-IMU) and a MMC markerless motion capture (MMC) system. First, five physiotherapists independently operated the iARAT-IMU across seven clinical routine assessment sessions at a rehabilitation clinic in Switzerland to quantify learning curves, setup times, and usability. Second, we conducted a preference study in which the same therapists used both, the IMU- and MMC-system, during a standardized drinking task and completed quantitative and qualitative usability assessments focusing on system preference and underlying reasons. Results show that therapists rapidly learned to operate the tablet application for scoring the iARAT; however, the IMU system added approximately 11 min of setup time and sometimes required assistance. In contrast, the MMC workflow required approximately 2 min of additional time - well within the 5-minute maximum indicated a priori by therapists as acceptable for clinical routine and received consistently higher usability ratings. Most therapists preferred this approach due to greater efficiency and reduced patient burden. These findings highlight important design considerations for future digital assessment tools and indicate that MMC systems may offer a more feasible pathway toward routine biomechanical data collection for upper-limb assessments in clinical neurorehabilitation.

## Introduction

Measuring movement kinematics during neurorehabilitation offers substantial benefits for both clinical practice and research [1–4]. Objective measurements in clinical routine allow precision rehabilitation, where therapy can be customized to individual patients based on detailed movement profiles and movement quality metrics rather than coarse ordinal scales [5]. Furthermore, systematic kinematic recording in clinical routine at scale facilitates big-data approaches for research to understand recovery trajectories, and provide precise evidence of patient progress over time [6].

However, even in technologically progressive clinics, the adoption of new rehabilitation technology remains limited [7] and skewed toward therapeutic devices, with measurement and assessment tools seeing considerably less uptake [8, 9]. Quantification of upper limb sensorimotor function through movement measurement remains rare to non-existent in clinical practice, even as clinicians and researchers increasingly call for such tools [2]. The feasibility of capturing movement data in clinical routine depends largely on the accuracy and usability of the measurement system as it needs to provide accurate measures to add clinical value while keeping the effort of data collection minimal for clinical staff during daily rehabilitation.

Marker based optical motion capture (OMC) provides highly accurate kinematics and can be used to precisely assess upper limb movement quality in people with stroke as shown by Alt-Murphy et al. [10]. However, besides its high financial cost, its clinical application is severely limited by its complexity, requiring training in software operation and marker placement, as well as the high time effort of calibration and marker placement. Consequently, in practice, marker-based optical motion capture remains limited to laboratory environments, serving only as an occasional supplement to clinical decision-making rather than an integrated component of routine neurorehabilitation.

Alternative measurement systems, such as inertial measurement units (IMUs) [11] and markerless motion capture (MMC) systems [12], have demonstrated sufficient accuracy in validation studies under controlled conditions and are promising candidates to bridge the gap; however, their adoption in daily clinical practice still remains limited due to their novelty and still remaining setup and calibration procedures. Although ease of use for these systems is expected to be higher than for OMC, their clinical usability from a routine data collection perceptive has not been studied yet. Therefore, assessing clinical usability with a focus on the practicality and effort required to acquire movement data in real rehabilitation settings is essential for determining which systems hold true translational potential and how systems might need to be adapted to increase in clinic usability and hence increased clinical adoption.

The most straightforward way of integrating these measurement systems into clinical routine is to instrument existing assessments which are already done on a routine basis in clinic, avoiding the introduction of a new protocol. The Action Research Arm Test (ARAT), a 19-item measure aimed at assessing functional performance of the upper extremity through observational means, is widely used in neurorehabilitation clinics. The instrumented Action Research Arm Test (iARAT) project represents such a translational effort. The core idea is to instrument the ARAT without changing its clinical assessment protocol, while the additional sensors provide kinematic outcome measures. The iARAT-IMU extends the conventional ARAT by integrating wearable motion sensors (IMUs) that stream motion data to a hand-held tablet, from which therapists can start and stop measurements and enter scores, additionally replacing pen and paper scoring. Despite successful early in-clinic deployment in a swiss neurorehabilitation clinic, two limitations have persisted: (1) measurements still depend on the support of research staff for technical setup, and (2) reliable kinematic reconstruction using IMUs remains challenging in clinical environments, which limits the set of outcome measures. As a result, although therapists are familiar with the scoring app, they did not conduct the full measurement process independently in clinical routine and lack a rich set of outcome measures. Hence, the first aim of this study is to evaluate usability of the IMU based iARAT when fully operated by therapists in clinical routine to assess whether data collection routine is satisfactory for therapists, which would provide strong justification to increase research efforts on reliable reconstruction of IMU kinematics in real-world settings.

Second, given the known limitations of IMU-based systems and recent advances in MMC systems, the second aim is to evaluate the usability of a MMC system for movement data collection, directly comparing it to the IMU-based approach with a focus on data collection workflow from the therapists’ perspective and their reasoned preferences. MMC eliminates the need for physical marker placement on the person by instead using computer vision algorithms detecting keypoints such as the joints of a person from RGB-Video. Mutli camera setups increase robustness against occlusion and allow for full 3D-biomechanical reconstruction with high accuracy [12]. Accuracy and reduced setup burden by reduction of marker placement makes MMC a promising alternative. Consequently, comparing both systems, IMU- and MMC-based for data collection in clinical routine will give direction on where research and development focus should be put next to finally bridge the gap of in-clinic motion data collection by providing an accurate measurement tool with high usability in clinical routine.

## Methods

### Overview

This study consisted of two parts. First, we evaluated the usability of the iARAT-IMU system when fully operated by physiotherapists during routine clinical assessments. During these same sessions, the MMC system recorded simultaneously to assess kinematic reconstruction quality in a real clinical environment. These recordings stem from the original data collection and did not involve a separate measurement session. Setup and calibration of the MMC system were performed by a researcher rather than the therapists to avoid imposing a double burden during clinical routine. This setup included syncing the tablet with the camera system through a visual cue shown on the tablet and captured by the cameras.

Second, we conducted a preference study, directly comparing an IMU-based system with a MMC system with the same therapists outside of clinical routine. Part one of the study was performed in clinical routine, since it was already integrated, whereas part two was performed with the same therapists in a separate session outside of clinical routine to get some initial data comparing IMU and MMC workflows without hindering clinical routine. For the second part, three repetitions of the standardized drinking task (a well studied task to assess upper limb movement quality in people with stroke [10]) instead of a full ARAT were used as motor task to reduce the measurement time and making time cost comparable to the drinking task protocol reported by Alt-Murphy et al. [10] which applied OMC and took 10-15 min. Both studies were performed at the neurorehabilitation clinic Cereneo and were approved via a waiver by the local Ethics Committee (EKNZ, BASEC number: Req-2024-01069).

**Fig. 1:**
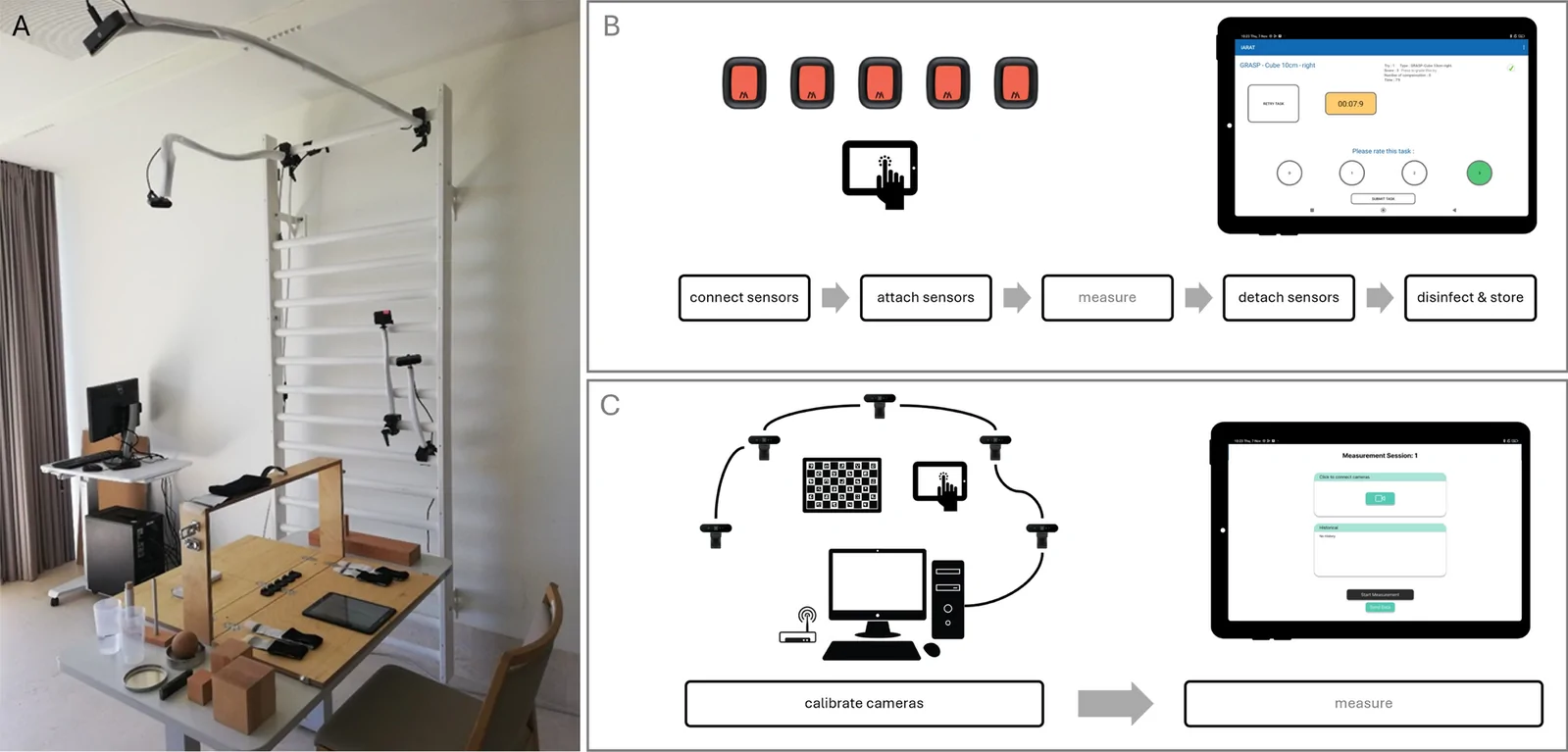
**A**: Setup of iARAT in clinic with IMU-based and MMC-based tool in place. **B**: Schematic overview of IMU-based tool with screenshot of tablet app and required phases to conduct measurement. **C**: Schematic overview of MMC-based tool with screenshot of tablet app and required phases to conduct measurement

### Part I: iARAT-IMU usability study

*Materials.* The iARAT system comprises the conventional ARAT materials, an Android tablet with a custom scoring application, and five Movella Dot IMUs with attachment materials (clips and Velcro straps; see Fig. 1A, B). A stopwatch was used by the researcher to record execution time and document whether assistance was required (see *Outcome Measures iARAT*, Supplementary Material). Several paper-based questionnaires were used. A custom questionnaire, administered after each session, used visual analog scales to assess satisfaction with hardware and software, perceived improvement in using the iARAT system, whether the session was completed without issues, perceived difficulty of sensor attachment (easy, medium, hard; which can vary with patient mobility) and participants’ attitudes toward adopting new technologies (see *Questionnaire iARAT*, Supplementary Material). The System Usability Scale (SUS) [13] was administered after the first and last sessions, with separate ratings for software (tablet application) and hardware (IMUs; see *Usability Questionnaire iARAT*, Supplementary Material).

*Participants.* All five physiotherapists who routinely perform upper-limb assessments at the clinic participated in the study, conducting assessments with sub-acute stroke patients during their scheduled clinical sessions (impairment range: severe to mild; mean ARAT score: *39 ± 12*, range 18–57). A researcher with expert knowledge of the iARAT system was present throughout each session to record timing data, collect questionnaire responses, and provide assistance only when required, documenting the reason and affected step for each instance. Prior to the clinical study, five researchers with expertise in operating the iARAT performed timed sessions to establish reference performance times.

*Procedure.* Each physiotherapist completed seven iARAT sessions, each comprising the following phases: (1) system preparation (connecting IMUs and preparing the tablet), (2) IMU placement on the patient, (3) administration of the ARAT, (4) sensor removal, and (5) system cleanup (disinfecting straps and storing IMUs and tablet). Phase 3 was excluded from temporal evaluation as ARAT duration depends on patient sensorimotor function rather than the measurement system. Therapists completed the questionnaires at the end of each session.

*Outcome Measures.* Quantitative measures included: (i) per-phase and total execution time; (ii) requirement of assistance (yes/no), including reason and phase); (iii) attainment of expert mode (yes/no)—a therapist was considered to have reached *expert mode* when three consecutive sessions were completed within the expert time threshold ($T_{\text {expert}} = \mu _{\text {expert}} + 2\sigma _{\text {expert}}$, where $\mu _{\text {expert}}$ and $\sigma _{\text {expert}}$ denote the mean and standard deviation of completion times of the five prior recorded iARAT experts) without requiring assistance. The expert threshold was established by researchers mimicking clinical use as closely as possible, serving as a benchmark for independent and efficient operation rather than a normative clinical standard; (iv) System Usability Scale (SUS) scores, collected separately for the tablet application and sensor system after the first and last sessions; and (v) session-specific scores on the custom questionnaire assessing satisfaction, perceived improvement, and sensor attachment difficulty. Qualitative feedback was collected as free-text comments after each session and analyzed thematically (see *Custom Questionnaire*, Supplementary Material).

### Part II: preference Study-IMU vs. MMC

*Materials.* The IMU-based technical setup was identical to Part I. The MMC setup consisted of: (1) three fixed, wall-mounted Logitech webcams, (2) a computer controlling and recording the cameras, (3) a tablet with a custom application for scoring and remote control of the recording software via local network, and (4) a calibration board (A1 sized ChArUco board; see Fig. 1). As upper-limb assessments are always conducted at the same table in the clinic, the cameras were permanently mounted around this table and did not require repositioning between sessions. A custom questionnaire using a 7-point Likert scale was administered to evaluate and directly compare both systems across key domains: ease of use, setup time, ease of learning, and technical robustness during data collection (see *Questionnaire*, Supplementary Material). A semi-structured interview with nine questions assessed system preference and underlying reasons (see *Semi-Structured Interview*, Supplementary Material).

*Participants.* Four of the five physiotherapists from Part I participated, each in an individual session; the fifth therapist could not participate due to scheduling conflicts. A researcher supervised each session and recorded data, and an able-bodied volunteer served as the measured subject.

*Procedure.* Prior to testing, all participants completed a pre-study questionnaire to gather requirements and expectations regarding system usability, including preferred end-user device (tablet or computer), willingness to integrate such systems into clinical routine, and maximum acceptable additional setup time (see *Pre-Study Questionnaire*, Supplementary Material). Each participant attended a one-hour session to test both the IMU-based and MMC systems. Testing order was counterbalanced (two participants started with IMU, two with MMC). Both systems followed an identical protocol: system setup followed by three repetitions of the standardized drinking task as described by Alt Murphy et al. [10]. For the IMU-based system, the setup procedure was identical to Part I. For the MMC system, setup involved initiating calibration on the tablet and presenting the ChArUco board to the fixed cameras for approximately one minute. Both systems were controlled via custom tablet applications designed specifically for the drinking task, distinct from the iARAT application used in Part I. To minimize instructor bias, all participants viewed the same video tutorial on system operation, which remained available for reference throughout the session. All steps were timed, and questionnaires were completed after testing both systems, followed by the semi-structured interview.

*Outcome Measures.* Quantitative measures included: (i) pre-study questionnaire responses, including maximum acceptable setup time; (ii) scores on the custom questionnaire for both IMU and MMC systems; and (iii) completion time per system. Qualitative data were collected via semi-structured interviews assessing system preference and underlying reasons, and analyzed thematically (see *Semi-Structured Interview*, Supplementary Material).

## Results

### Part I: iARAT-IMU

**Fig. 2:**
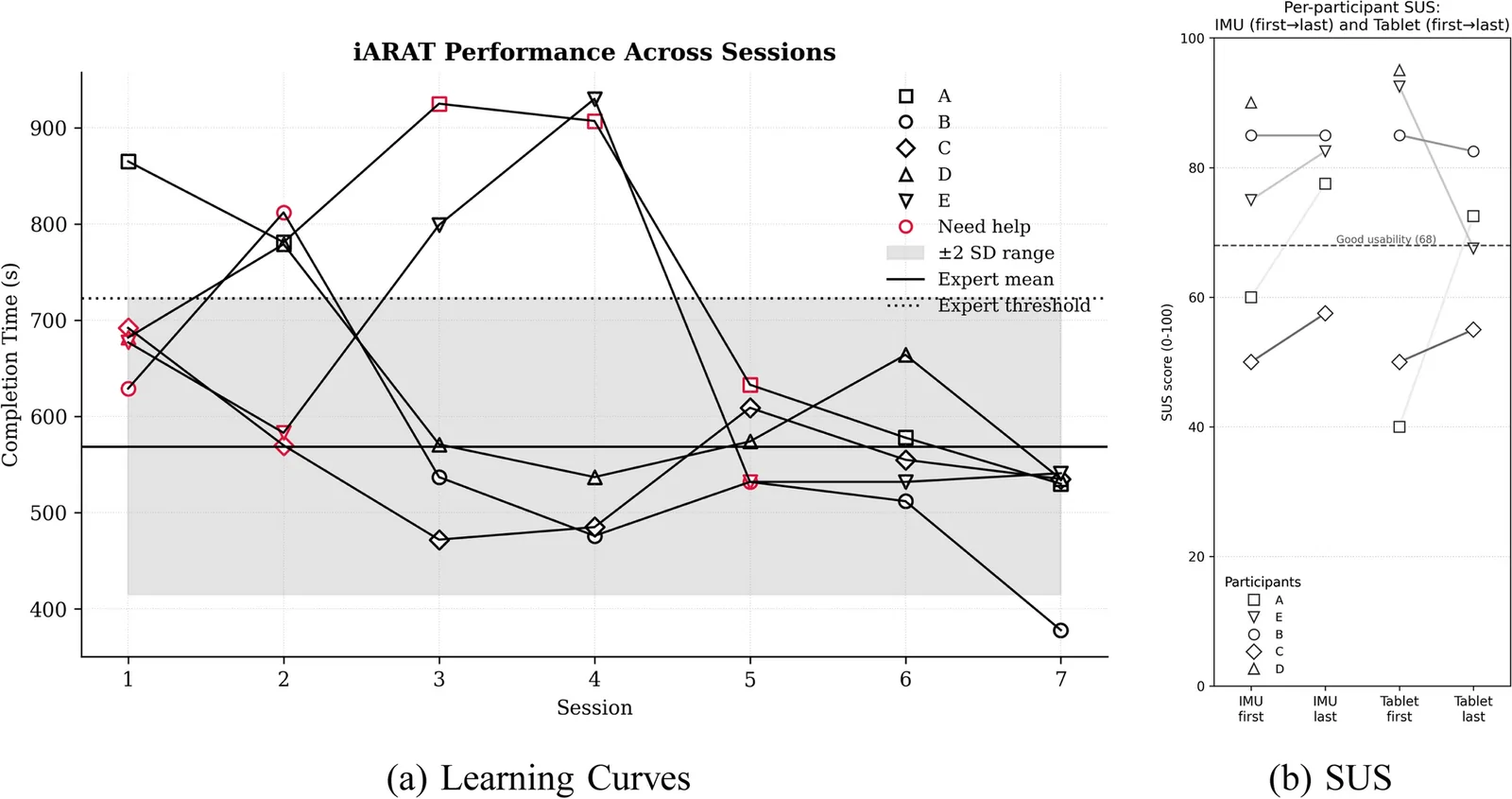
Results of in-clinic IMU-based iARAT usability study. **a** Learning curves per participant showing completion time in seconds, with indication of required help, elevated perceived difficulty per session, and expert reference times (mean and 2 SD). Per phase duration in detail in supplementary part (mean times (SD): (1) Connection of IMUs: 187 s (73 s), Attaching IMUs: 165 s (78 s), Taking IMUs off: 55 s (31 s), Putting IMUs away: 221 s (79 s), see A2). **b** Results of Systems Usability Scale (SUS) differentiating between usability of tablet app and sensor system on first and last session. Therapist D could not complete the final session due to scheduling conflicts, hence data point missing

*Quantitative.* The average additional time across all sessions and therapists for one iARAT session was 10:24 min, approximately 1 min slower than the expert group mean of 9:29 min (SD: 1:17). The expert time threshold, defined as mean + 2SD, was 12:03 min. Across all therapists, system cleanup took the most time, with approximately 4 min spent removing IMUs from clips and disinfecting straps. Both system preparation (IMU connection and tablet setup) and sensor placement on the patient required approximately 3 min each, whereas sensor removal was the fastest phase at roughly 1 min (see Fig. A2 in Supplementary Material for detailed distributions). All participants showed a clear decrease in completion time across the seven sessions. This learning effect is visible in Fig. 2a and reflected numerically in Table 1, where mean duration decreased from 11:49 min in Session 1 to 8:24 min in Session 7. Sessions in which therapists required assistance (11/35 sessions; 31%) or reported increased difficulty attaching sensors due to patient condition (4/35 sessions; 11%) were noticeably longer. The longest recorded session (Therapist A, Session 3) lasted 15:25 min and involved both factors. In 9 of the 11 instances requiring assistance, help was needed during the IMU–tablet connection step, typically due to connectivity issues (see Fig. A2). In the remaining two instances, assistance was needed during cleanup when therapists struggled to remove IMUs from clips. All therapists required help in at least one session, usually early in the training phase. Based on the predefined expert criteria - three consecutive sessions completed without assistance and within the expert time threshold - four of five therapists reached expert mode. The fifth therapist completed two consecutive expert-level sessions (Sessions 6 and 7) and was therefore close to reaching this criterion. Therapist E achieved expert mode after Sessions 2–4 but required assistance again in Session 5. By the final session, all therapists achieved completion times below the mean expert reference time without requiring assistance, demonstrating consistent learning of the iARAT-IMU system. On average, the IMU system achieved a SUS score of 72 points on the first session and 76 points on the last session. The tablet application scored 73 points on the first session and 69 on the last. Scores varied across therapists; Therapist C, who reported the lowest openness to new technology (neutral attitude; see Fig. A2), consistently scored lower than other participants, with scores around 50 points across all entries (see Fig. 2b). SUS scores for IMUs slightly improved over time while scores for the tablet application slightly decreased, with both systems exceeding the threshold for acceptable usability (SUS *≥ * 68; [14]). However, scores in the 69–76 range are modest for a system intended for daily clinical use, and the slight decline in tablet application scores over time reflects growing awareness of specific limitations through repeated use, such as the absence of back navigation and limited flexibility for re-doing items, as supported by the qualitative feedback.

The MMC system, recorded in parallel during the Part I sessions, yielded a mean reprojection error of approximately 12 px across all sessions, comparable to results obtained under controlled laboratory conditions [12]. Visual inspection of kinematic overlays confirmed anatomically plausible reconstructions across patients, including those with more severe impairments.

**Table 1:** Session durations (min: sec) per therapist and reaching of expert status (Expert status: 3 consecutive sessions without help needed and within expert time (12:03 min))

Participant	S1	S2	S3	S4	S5	S6	S7	Mean	Status
Therapist A	14:25	13:01$^{\dagger }$	15:25*${}^{\dagger }$	15:07*	10:33*	09:38	08:50	12:11	2/3
Therapist B	10:29*	13:32*	08:57	07:56	08:52	08:32	06:18	09:14	Expert
Therapist C	11:32$^{\dagger }$*	09:30$^{\dagger }$*	07:52	08:05	10:09	09:15	08:55	09:20	Expert
Therapist D	11:22*	12:59*	09:31	08:57	09:34	11:04	08:55	10:20	Expert
Therapist E	11:17*	09:43	13:19$^{\dagger }$	08:51	15:30*	08:52	09:01	10:56	Expert
**Mean (session)**	**11:49**	**11:45**	**11:01**	**09:47**	**10:56**	**09:28**	**08:24**	**10:24**	

Bold values indicate mean times over all therapists for each session. *: Help needed (11/35 sessions). $^{\dagger }$: increased perceived difficulty attaching sensors to the patient due to patient’s condition (5/35 sessions)

*Qualitative.* The iARAT tablet application was generally perceived as intuitive and easy to use, especially once the sensors were connected, although this initial step required some learning. Therapists highlighted automatic timing, immediate score visualization, and reduced administrative workload - particularly eliminating manual transfer of scores to digital clinic systems - as key advantages over the traditional paper-based ARAT. However, some usability limitations were noted, including the absence of back navigation for error correction and reduced flexibility in redoing items and taking custom notes compared to pen-and-paper scoring. Setup and preparation time, particularly related to sensor attachment, was identified as a significant contributor to overall assessment duration. For patients with more severe sensorimotor impairments, attaching sensors required extra effort and time, occasionally causing discomfort. Once properly attached, IMUs were often described as non-intrusive during task execution; however, issues persisted during some assessments, including loose straps and sensors slipping or falling off. Regarding the clinical value of IMU integration, therapists acknowledged the potential of kinematic data for quantifying movement quality parameters such as trunk compensation, tremor, and movement speed, and expressed interest in a richer set of outcome metrics in the future, potentially including hand and finger movements. Nevertheless, the current implementation was considered insufficient for routine clinical use due to the sensor attachment burden and technical synchronization problems; the added clinical value of current outcome measures (trunk compensation and end-effector smoothness) did not justify the extra effort. If retaining an IMU-based system, most therapists expressed a preference for a hybrid approach, using the iARAT for standard assessments while retaining the paper-based version for patients with severe impairments or in time-constrained settings. In summary, the digital scoring application was perceived as an improvement over traditional pen-and-paper scoring, primarily due to time savings in clinical routine. Therapists strongly recommended allowing greater in-application flexibility for repeating tasks, taking custom notes, and aligning the user interface more closely with the traditional ARAT scoring sheet. While the potential of movement measurement and associated clinical insights was widely acknowledged, the current trade-off between the extra effort required to use IMUs and the outcome measures provided was not sufficient to justify integration into clinical routine.

### Part II: preference study

**Fig. 3:**
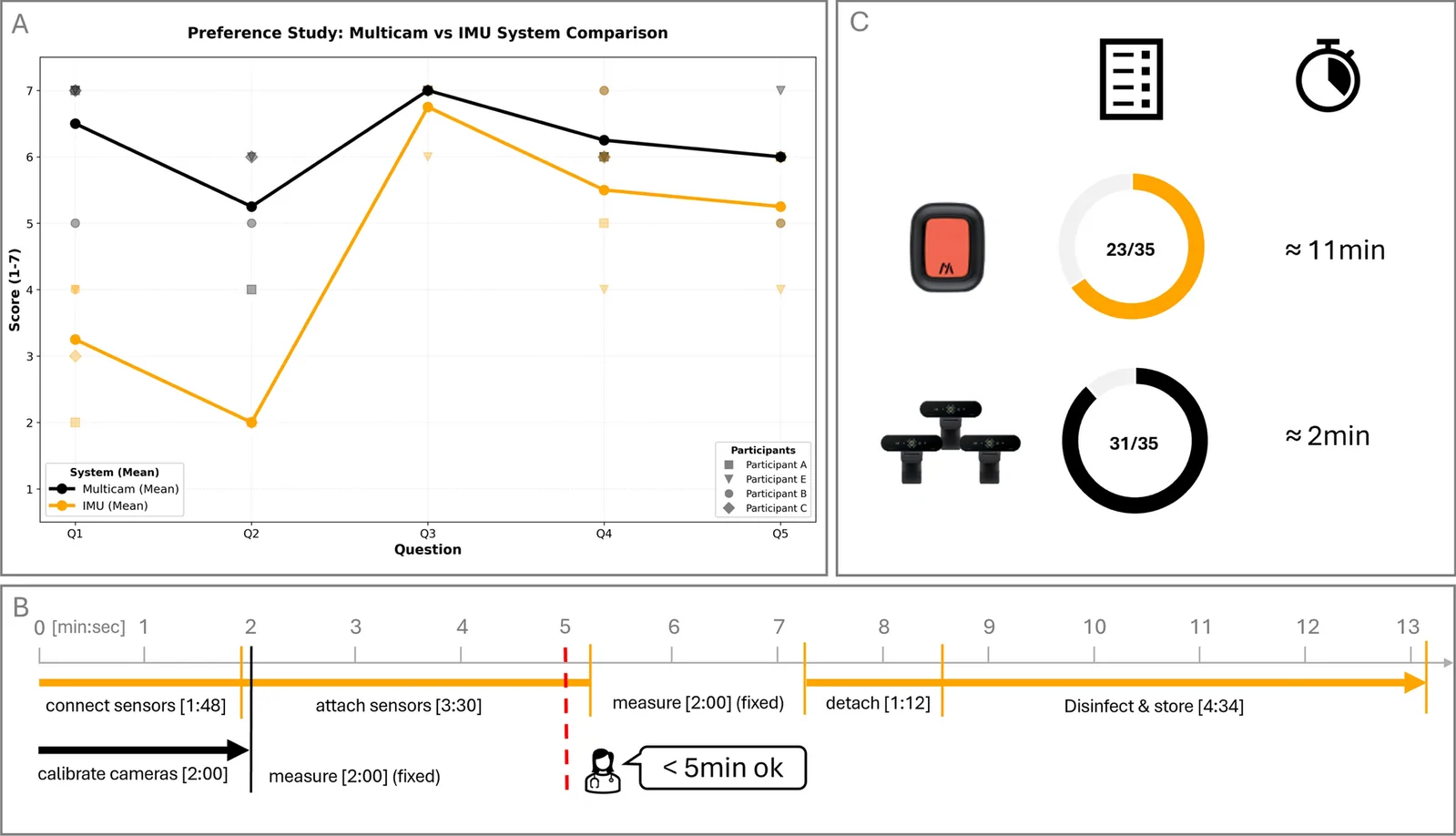
**A**: Preference study questionnaire results (1: fully disagree, 7: fully agree). (Q1): The setup process was fast, (Q2): The setup process was not cumbersome. (Q3) The setup process was easy to learn and to remember. (Q4) I did not have technical difficulties using the system. (Q5) Overall, I am content with the assessment workflow. **B**: mean completion time over all therapists per tool with 5 min bar marking the acceptable extra time by therapists, **C**: Overview: total questionnaire score and total average time per tool

*Pre-preference study requirements.* Prior to the study, four of five therapists indicated that 5 min was the maximum acceptable additional time to instrument an assessment in clinical routine; one therapist indicated 10 min. All emphasized that less time was preferable, and 5 min was therefore established as the critical threshold for subsequent evaluation. All therapists preferred using a tablet, or a tablet remotely controlling a computer, over using a computer directly; the MMC system was designed accordingly with a tablet as the end-user device. One therapist could not participate in the preference study due to scheduling conflicts, reducing the sample to four therapists.

*Quantitative.* The IMU-based system added approximately 11 min of additional time on average (see Fig. 3), comparable to the in-clinic iARAT mean (10:24 min; see Table 1) and slightly longer than completion times after the learning phase in Part I, reflecting a slight learning curve for the new application interface. All sessions were conducted without external support, except for one instance where a therapist wearing a finger cast required assistance removing IMUs from clips. The setup process for the MMC system took 2 min on average, with minimal variation between therapists (less than 10 s). On the custom questionnaire, therapists scored the IMU-based system 23 out of 35 points on average, compared to 31 out of 35 for the MMC system (see Fig. 3). The largest differences were observed for Question 1 ("The setup process was fast") and Question 2 ("The setup process was not cumbersome"), reflecting the additional effort required to set up and disassemble the IMU system compared to the camera system. Both systems scored highly on ease of learning and recall (6–7 out of 7 for Question 3). Both systems also achieved high scores (approximately 5–6 out of 7) for Question 4 ("I did not have technical difficulties using the system") and Question 5 ("Overall, I am content with the assessment workflow"), with slightly higher values for the MMC system. For both systems, technical difficulties were related to instabilities in wireless device connections. For the MMC system this was a minor and isolated issue related to shared Wi-Fi network traffic, which could be mitigated by using a dedicated private network. For the IMU system, connectivity issues were more frequent, with the Bluetooth connection between tablet and sensors not always established reliably at the start of sessions. While no undetected connection loss occurred during measurements in this study, sensors occasionally lost connection when dropped, which was noticed when they were picked up again. As such a loss could in principle go undetected, future versions could mitigate this risk by triggering a warning whenever connection to a sensor is lost. Connection quality in general is largely dependent on the SDK provided by the IMU manufacturer and therefore outside the direct control of the application. In summary, the MMC system scored higher primarily due to ease of use and reduced setup time, meeting the 5-minute threshold established a priori (2 min) whereas the IMU-based system did not (11 min).

*Qualitative.* Three of four therapists preferred the MMC system, while one remained undecided. The primary factors influencing preference were time efficiency, ease of use, and patient comfort. Most therapists emphasized the importance of minimizing setup time to avoid disruptions within their constrained schedules. The undecided therapist attributed their indecision to extensive experience with IMUs but limited familiarity with camera-based assessments. Most therapists reported that patients generally did not object to being filmed. However, they found the IMU setup cumbersome, particularly for patients with limited upper-limb mobility, as the Velcro bands securing the trunk and upper arm sensors require lifting the arm to create sufficient space for wrapping, which was challenging and occasionally caused discomfort for patients with reduced mobility or pain. This added difficulty was considered a significant drawback, especially for patients already facing movement challenges. Although this study focused primarily on data collection effort, all therapists articulated a clear trade-off between collection effort and the added value of outcome measures for clinical practice. Given that different systems are currently used within the clinic, therapists expressed a desire for a harmonized tool that could integrate seamlessly with existing instruments—for example, a modular system combining sensor- and vision-based assessments within a single platform. Three of four therapists expressed interest in an option to incorporate additional wrist sensors for assessing movement smoothness when using MMC. Regarding outcome measures and their presentation, therapists suggested that the tool should include predefined metrics for kinematic assessment, with the option to access more detailed metrics and longitudinal data as needed. This approach would enable more streamlined and consistent assessments across patients. Additionally, therapists indicated that the tool should be intuitive and require minimal training. The importance of reliability—specifically, a tool that does not require frequent recalibration—was emphasized, as this would reduce cognitive load during busy clinical sessions. In summary, the MMC system was preferred due to its ease of use and minimal time requirements. Therapists expressed interest in a harmonized system allowing modular selection of measurement modalities (camera, IMU, or combination) within a single integrated platform. Time efficiency, robustness, patient comfort, and ease of use were the factors most frequently cited as essential for seamless integration into clinical routine.

## Discussion

This study is the first to evaluate and compare the usability of IMU-based and MMC systems for collecting biomechanical movement data during upper-limb assessments in clinical neurorehabilitation, identifying reasoned preferences from the therapists’ perspective. Initial in-clinic evaluation of the tablet and IMU-based system demonstrated that therapists learned to operate the tool rapidly, achieving expert-level performance regarding administration time and independent use within several sessions. While replacing pen-and-paper scoring with a digital tablet application was largely perceived as an improvement in clinical workflow - eliminating manual transfer of scores to electronic health records - adding sensors to the assessment presented limitations, primarily the additional time required for sensor setup (approximately 11 min). This prompted the introduction of a MMC system as alternative, also operated via tablet, allowing direct comparison of both systems to systematically evaluate workflow differences and obtain reasoned feedback on system preference. When directly comparing the two systems, therapists preferred the MMC approach, primarily due to reduced setup time (approximately 2 min versus 11 min for IMUs) and ease of use-specifically, not requiring instrumentation of the patient, which occasionally caused discomfort. This difference is particularly notable given that therapists were already experienced with the IMU system whereas the MMC system was new to them, indicating that the bottleneck lies in the workflow itself rather than operator proficiency. With therapists indicating a maximum acceptable additional time of approximately 5 min, the current IMU-based approach clearly exceeds this threshold. Notably, this was observed even at a clinic characterised by high technology acceptance and a favourable therapist-to-patient ratio, representing a best-case scenario for adoption. Although optimized attachment strategies could potentially reduce IMU setup time, the MMC system already meets this requirement. As the reconstruction algorithm can correct small calibration errors, calibration time could be further reduced in the future by ensuring stable camera configurations and incorporating automatic calibration checks that only prompt recalibration when necessary. While this study primarily disentangled data collection effort from outcome metrics, therapists emphasized a clear trade-off between willingness to invest time and the quality of outcome measures obtained. The parallel MMC recordings obtained during Part I provided an initial data quality check in a real clinical environment, with reprojection error and visual overlay inspection yielding results comparable to in-lab settings, providing encouraging evidence that reconstruction quality is maintained in clinical routine. Nevertheless, in clinical routine, time constraints remain paramount regardless of outcome quality, which likely explains why marker-based optical motion capture has not achieved translation from laboratory to clinic despite requiring only 10–15 min of preparation time for an instrumented upper-limb assessment [10]. This interpretation is supported by the majority of therapists indicating 5 min as an upper limit for acceptable additional time, with strong emphasis on minimizing this burden further. These results suggest that if data collection time cannot be reduced below 5 min, systems will likely not be adopted in daily routine. Consequently, improving kinematic reconstruction algorithms for IMUs to obtain richer outcome metrics will not facilitate routine clinical application until data collection time meets this requirement. Notably, this study did not include an additional sensor-to-segment calibration step, which, in the absence of automatic calibration algorithms, could add further time and effort to IMU-based workflows. Since previous studies demonstrate that full kinematic reconstruction using MMC with the calibration protocol employed here is feasible for upper-limb assessments [12], with potential extension to hand and finger tracking demonstrated both within the same MMC framework [15] and in other work showing promising accuracy for markerless hand and finger tracking [16], and given the superior data collection usability demonstrated in this study, there is strong justification to prioritize development of the MMC system for daily clinical use. Future work should further evaluate its robustness and usability in real-world clinical settings with patients and clinicians. Subsequently, outcome measures should be incorporated into usability evaluations to assess the complete tool, contextualizing data collection effort with the added clinical value of novel kinematic metrics [17]. This study has several limitations. First, the calibration of the MMC system by therapists was evaluated only in a controlled setting with an able-bodied volunteer, not in clinical routine with patients. However, the calibration process is independent of the subject’s functional status, and data quality checks on recordings obtained during in-clinic measurements confirm that the system maintains high reconstruction quality in clinical environments. Second, the study was conducted at a single rehabilitation clinic characterized by high technology acceptance and a favorable therapist-to-patient ratio. Results could therefore differ in other clinical settings with greater time pressure or less technology experience, likely with even more stringent time requirements. The exact maximum acceptable setup time is therefore clinic-specific, depending on caseload and time pressure, plausibly ranging from less than a minute in the most time-constrained settings up to the 5 min observed here. This reinforces that setup time should be minimized as far as possible rather than treated as a fixed target, and that the current IMU-based workflow does not meet clinical needs even under these optimal conditions.

## Conclusion

This is the first study to evaluate the usability of collecting biomechanical movement data during upper limb assessments for precision neurorehabilitation in clinical routine, comparing IMU-based and MMC systems and identifying reasoned preferences from the therapists’ perspective. Results demonstrate a clear advantage of the MMC approach over the IMU-based approach for data collection in clinical routine upper limb assessments, primarily driven by reduced instrumentation time—approximately 2 min for MMC compared to 11 min for IMUs—where 5 min was the upper acceptable limit indicated by the majority of therapists. These findings provide clear direction to prioritize the development and integration of MMC over IMU-based systems for clinical routine upper limb assessments. This technology holds promise for enabling biomechanical data collection at scale in clinical routine while keeping the burden on therapists minimal.

## Additional file

Supplementary file 1.

## Data Availability

The dataset supporting the conclusions of this article is included within the manuscript and supplements. Raw data is available at reasonable request from the corresponding author, as the transcripts contain personal identifiable information that preclude public availability.
