# Supplementary material for: Towards routine biomechanical data collection in stroke rehabilitation: a usability comparison of IMU and markerless motion capture systems for functional upper-limb assessments
Source: J Neuroeng Rehabil. 2026 Jul 7;23:286. doi: 10.1186/s12984-026-02075-7 (PMC13625311; doi:10.1186/s12984-026-02075-7)
Supplement: Supplementary file 1 [file 12984_2026_2075_MOESM1_ESM.pdf]

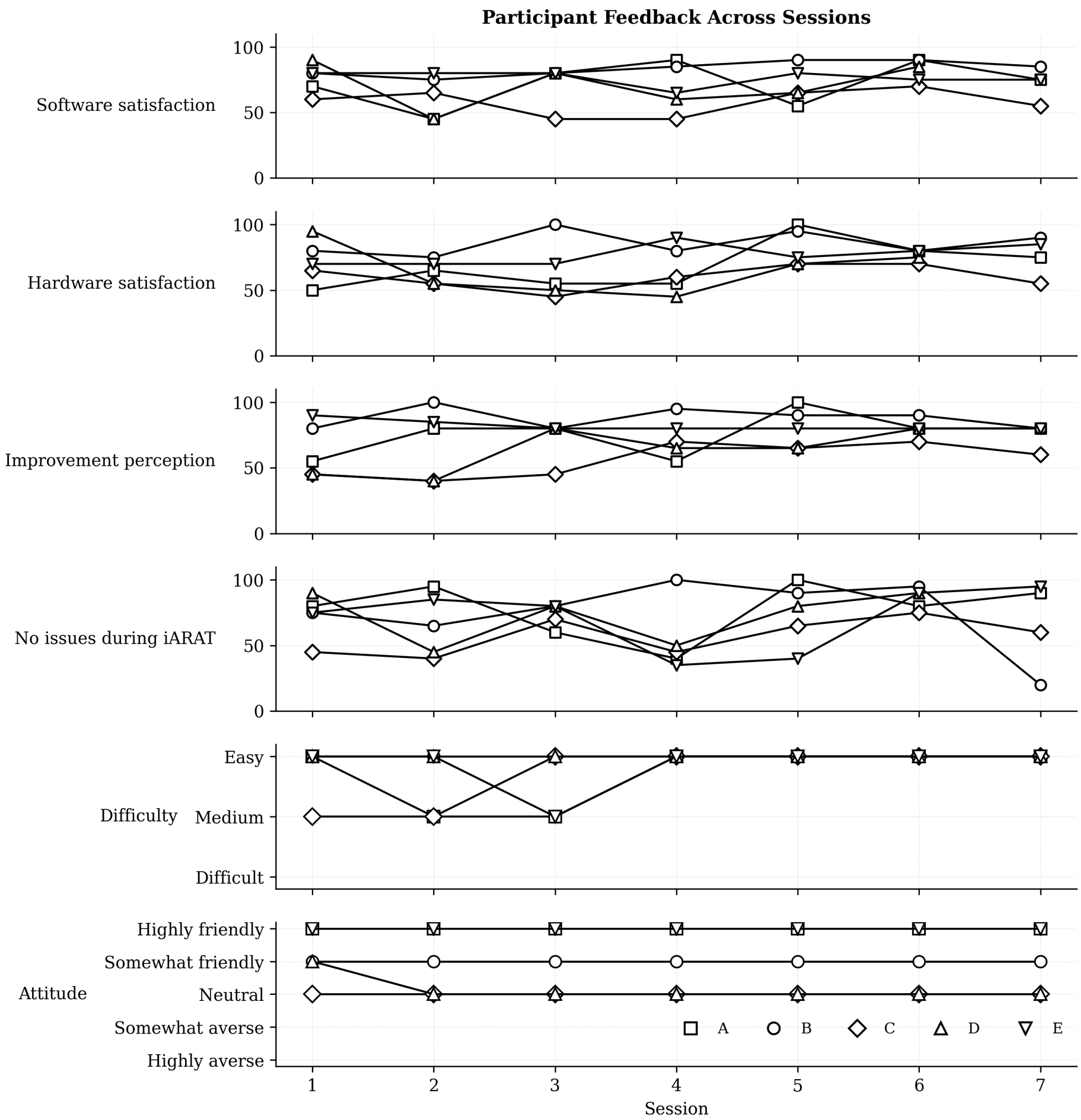

Fig. A1: SmallQ

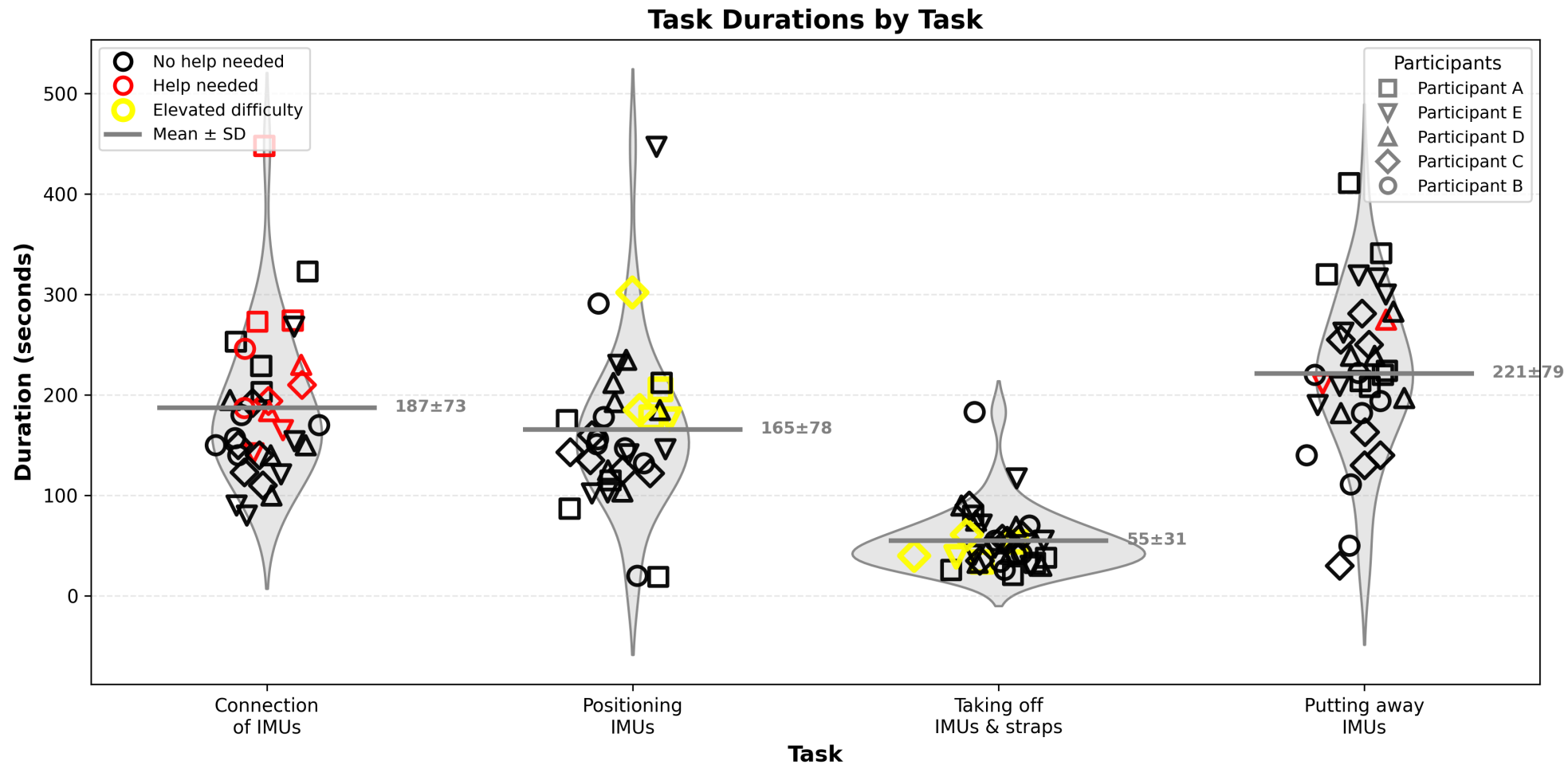

Fig. A2: Execution time per phase per therapist across seven iARAT-IMU sessions in clinical routine. Sessions requiring assistance (red) and sessions with elevated perceived difficulty attaching sensors due to patient sensorimotor impairment (yellow) are color-coded.

Participant	P1	P1	P1	P2	P2	P2	P3	P3	P3	P4	P4	P4	P5	P5	P5
Connecting	201	210	296	143	142	126	186	126	156	175	119	117	136	108	99
Putting	92	93	129	139	142	119	115	117	100	140	130	127	137	110	113
Taking off	40	37	39	37	47	43	37	48	45	44	43	45	42	39	43
Storing	228	259	226	213	228	213	231	202	223	376	339	365	247	207	213
Total Time	561	599	690	532	559	501	569	493	524	735	631	654	562	464	468

Table A1: Expert reference times (in seconds) for iARAT session phases. Participants were instructed to work at a comfortable but efficient pace. Each participant completed three sessions. Mean total time: 9:29 min (SD: 1:17 min); expert time threshold (mean + 2SD): 12:03 min.

569

Outcome measures iARAT

To be filled out by the person doing the usability testing after each session

Task completion time

Fill in with the time needed for each step in seconds.

Task	Completion time [s]
1. Connection of IMUs and preparation of the Tablet	
2. Positioning of IMUs on the patient	
3. Recording of the scores in a digital format	
4. Taking IMUs and straps off the patient	
5. Putting away IMUs and Tablet	

Help

Complete the table according to if help was needed for the person carrying out the test.

Task	Help : **yes - no**	Help : **S – M - T**
5. Connection of IMUs and preparation of the Tablet		
6. Positioning of IMUs on the patient		
7. Recording of the scores in a digital format		
8. Taking IMUs and straps off the patient		
6. Putting away IMUs and Tablet		

Legend: *The letters correspond to which person is currently doing the different tasks of the iARAT.*

S - LLUI student ; **M** – mixed: Therapist and student together ; **T** - Therapist

Questionnaire iARAT

To be filled out by the person doing the iARAT after each session

Overall software satisfaction level – *Tablet (App)*

Please draw a vertical line at the most appropriate position on the scale.

0 ¦---¦ **100**

Comments :

Overall hardware satisfaction level - *IMUs*

Please draw a vertical line at the most appropriate position on the scale.

0 ¦---¦ **100**

Comments :

Improvement perception in doing the iARAT

Please draw a vertical line at the most appropriate position on the scale.

No ¦---¦ **Yes**

Comments :

iARAT was conducted without any issues

Please draw a vertical line at the most appropriate position on the scale.

Strongly disagree ¦---¦ **Strongly agree**

Comments :

Perceived difficulty of putting on the sensors

Please circle the most appropriate adjective :

- **Easy** - I can put on the sensors as if I was putting them on a healthy person
- **Medium** - I can put on the sensors with some effort and adjustments
- **Difficult** - I need to put in significant effort and make adaptations

Comments :

How would you describe your overall attitude towards adopting new technology in your daily life ?

(Answer ONCE at the beginning of the usability study)

Please circle the most appropriate adjective :

1. Highly averse

2. Somewhat averse

3. Neutral

4. Somewhat friendly

5. Highly friendly

Comments :

Usability questionnaire iARAT

To be filled out by the person doing the iARAT after the first and the last session

Questions about IMUs

This section only relates to the IMUs devices. Please answer accordingly and put a cross in the most appropriate box.

Questions	1 Strongly disagree	2	3	4	5 Strongly agree
1. I think that I would like to use this system frequently.					
2. I found the system unnecessarily complex.					
3. I thought the system was easy to use.					
4. I think that I would need the support of a technical person to be able to use this system.					
5. I found the various functions in this system were well integrated.					
6. I thought there was too much inconsistency in this system.					
7. I would imagine that most people would learn to use this system very quickly.					
8. I found the system very cumbersome to use.					
9. I felt very confident using the system.					
10. I needed to learn a lot of things before I could get going with this system					

Comments:

Questions about the tablet

This section only relates to the tablet. Please answer accordingly and put a cross in the most appropriate box.

Questions - 1st part	1 Strongly disagree	2	3	4	5 Strongly agree
1. I think that I would like to use this system frequently.					
2. I found the system unnecessarily complex.					
3. I thought the system was easy to use.					
4. I think that I would need the support of a technical person to be able to use this system.					
5. I found the various functions in this system were well integrated.					
6. I thought there was too much inconsistency in this system.					
7. I would imagine that most people would learn to use this system very quickly.					
8. I found the system very cumbersome to use.					
9. I felt very confident using the system.					
10. I needed to learn a lot of things before I could get going with this system					

Comments:

Custom questions

Tablet

Did you find the application intuitive? Yes, no and why?

How would you compare the time taken to score the ARAT on the tablet compared to the paper version?

How satisfied were you with the process of entering scores into the app compared to the paper version?

Overall, which method do you prefer for scoring the ARAT? Why?

What improvements would you suggest for the application to improve the scoring process compared to the paper version?

IMUs

Did the use of IMUs interfere with the natural flow of the iARAT test? Yes, no and why?

How do you rate the additional data provided by IMUs compared with the basic iARAT test alone?

How useful were the additional measurements provided by the IMUs in assessing upper limb function?

Overall, do you prefer the iARAT with or without the additional IMU data? Why?

What improvements would you suggest for the use of IMUs to improve the iARAT?

Pre-Preference Study Questionnaire

1. Would you prefer to conduct the assessment using a Multi-Camera-System or IMUs?

☐ Multi-Camera-System

☐ IMUs

2. Would you prefer to conduct the assessment on a tablet or a computer?

☐ Tablet

☐ Computer

3. Would you prefer to conduct the assessment on the computer alone, or would you like an additional tablet that mirrors the computer screen and with which you can also interact?

☐ Computer alone

☐ Computer and additional tablet

4. I believe that such a technical assessment tool could provide valuable new insights into the evaluation of patients.

1: Strongly disagree, 2: Disagree, 3: Somewhat disagree, 4: Neither agree nor disagree, 5: Somewhat agree, 6: Agree, 7: Strongly agree

1 2 3 4 5 6 7

5. I would be willing to integrate a technical assessment tool into the demanding daily routine of my clinic.

1: Strongly disagree, 2: Disagree, 3: Somewhat disagree, 4: Neither agree nor disagree, 5: Somewhat agree, 6: Agree, 7: Strongly agree

1 2 3 4 5 6 7

6. What is the maximum time you are willing to allocate for setting up the technology during your daily clinical routine for a measurement? [min]

Name: BASEC number: Req-2024-01069 Preference Study
Date:

571

Questionnaire

1: Strongly disagree, 2: Disagree, 3: Somewhat disagree, 4: Neither agree nor disagree, 5: Somewhat agree, 6: Agree, 7: Strongly agree

Multicam and IMU

1. The setup process was quick

Multicam	1	2	3	4	5	6	7
IMU	1	2	3	4	5	6	7

2. The setup process was not cumbersome

Multicam	1	2	3	4	5	6	7
IMU	1	2	3	4	5	6	7

3. The setup process was easy to learn and to remember

Multicam	1	2	3	4	5	6	7
IMU	1	2	3	4	5	6	7

Name: BASEC number: Req-2024-01069 Preference Study
Date:

Multicam and IMU

4. I did not have technical issues using the system

Multicam	1	2	3	4	5	6	7
IMU	1	2	3	4	5	6	7

5. I believe such a technical assessment tool could provide valuable new insights into the evaluation of patients.

Multicam	1	2	3	4	5	6	7
IMU	1	2	3	4	5	6	7

6. Overall I am content with the systems workflow

Multicam	1	2	3	4	5	6	7
IMU	1	2	3	4	5	6	7

Name: BASEC number: Req-2024-01069 Preference Study
Date:

Multicam specific

1. It is feasible to perform the calibration before the patient arrives.

Multicam	1	2	3	4	5	6	7

IMU specific

1. I think the patient would not mind the IMU setup process

IMU	1	2	3	4	5	6	7

Name: BASEC number: Req-2024-01069 Preference Study
Date:

Semistructured Interview

1. Which assessment tool did you prefer and why?
2. Is there anything you did not like about one or both assessment tools?
3. Which process would you prefer to integrate into your daily clinical routine?
4. Is there anything in the workflow of the assessment tools that you would like to change?
5. From your experience, do you think the patient would mind to be filmed or to wear IMUs? What do you think they would prefer?
6. The multicam tool can provide precise full upper limb kinematics but IMUs could provide more sensitive measures of end-effector smoothness. Does this information change your preference? Would you prefer a combination of both systems (multicam and IMUs on wrist) to obtain results from both systems?
7. If the patient does not want to be filmed, would you be willing to put in extra effort for IMU calibration to obtain IMU-based upper limb kinematics?
8. Are you satisfied with predefined outcome metrics or would you prefer to extract your own metrics?
9. Is there anything else you would like to let us know?

1. Which assessment tool did you prefer and why?

...
